# Pelvic Organ Prolapse Syndrome and Lower Urinary Tract Symptom Update: What’s New?

**DOI:** 10.3390/healthcare11101513

**Published:** 2023-05-22

**Authors:** Gaetano Maria Munno, Marco La Verde, Davide Lettieri, Roberta Nicoletti, Maria Nunziata, Diego Domenico Fasulo, Maria Giovanna Vastarella, Marika Pennacchio, Gaetano Scalzone, Gorizio Pieretti, Nicola Fortunato, Fulvio De Simone, Gaetano Riemma, Marco Torella

**Affiliations:** Obstetrics and Gynecology Unit, Department of Woman, Child and General and Specialized Surgery, University of Campania “Luigi Vanvitelli”, 80138 Naples, Italy; gmm9401@gmail.com (G.M.M.); marco.laverde88@gmail.com (M.L.V.); davidelett@gmail.com (D.L.); roberta.nicoletti81@gmail.com (R.N.); maria.nunziata5@libero.it (M.N.); diegodomenico1993@gmail.com (D.D.F.); mariagiovannavastarella@hotmail.it (M.G.V.); marikapennacchio@gmail.com (M.P.); drgscalzone@gmail.com (G.S.); gorizio.pieretti@unicampania.it (G.P.); nicola.fortunato@libero.it (N.F.); fulviodesimone65@gmail.com (F.D.S.); gaetano.riemma@unicampania.it (G.R.)

**Keywords:** overactive bladder, pelvic organ prolapse, lower urinary tract symptoms, urodynamics

## Abstract

(1) Background: This narrative review aimed to analyze the epidemiological, clinical, surgical, prognostic, and instrumental aspects of the link between pelvic organ prolapse (POP) and lower urinary tract symptoms (LUTS), collecting the most recent evidence from the scientific literature. (2) Methods: We matched the terms “pelvic organ prolapse” (POP) and “lower urinary tract symptoms” (LUTS) on the following databases: Pubmed, Embase, Scopus, Google scholar, and Cochrane. We excluded case reports, systematic reviews, articles published in a language other than English, and studies focusing only on a surgical technique. (3) Results: There is a link between POP and LUTS. Bladder outlet obstruction (BOO) would increase variation in bladder structure and function, which could lead to an overactive bladder (OAB). There is no connection between the POP stage and LUTS. Prolapse surgery could modify the symptoms of OAB with improvement or healing. Post-surgical predictive factors of non-improvement of OAB or de novo onset include high BMI, neurological pathologies, age > 65 years, and the severity of symptoms; predictors of emptying disorders are neurological pathologies, BOO, perineal dysfunctions, severity of pre-surgery symptoms, and severe anterior prolapse. Urodynamics should be performed on a specific subset of patients (i.e., stress urinary incontinence, correct surgery planning), (4) Conclusions: Correction of prolapse is the primary treatment for detrusor underactivity and for patients with both POP and OAB.

## 1. Introduction

In 1994, the term lower urinary tract symptoms (LUTS) was adopted to classify LUTS based on the presence of storage, voiding, and post-micturition symptoms [1]. The classification of LUTS included a wide range of clinical manifestations. Increased urinary frequency, nocturia, urinary urgency, and incontinence are included in the storage symptoms [1]. Slow/weak stream, hesitancy and terminal dribble, and immediate post-urination symptoms are included in voiding symptoms [2]. Following an evaluation of the patient’s LUTS, physical findings, the results of urinalysis, and other necessary investigations, physicians may frequently make an empirical diagnosis as the basis for initial management. Several studies in Europe and North America found a LUTS prevalence of more than 60% in men and women over 40 years [3,4], with a higher prevalence in women over 70 [5]. Pelvic organ prolapse (POP) is defined as the herniation of the anterior vaginal wall, posterior vaginal wall, uterus, or vaginal apex into the vagina [6] and is commonly related to complex symptoms such as LUTS [7]. Even if a vast number of women over 50 is affected by POP, only 20% exhibit symptoms [7]. However, according to population projections from the United States Census Bureau, the number of patients who will develop POP is expected to increase by 46%, from the current 3.3 million to 4.9 million by 2050 [8]. To date, LUTS with POP are common but inconsistently reported, and there are few data on this relationship’s incidence. Obstructive voiding is most frequently associated with POP [9]. Patients with isolated posterior POP should be examined for anorectal or bulging symptoms and LUTS [10]. The low severity of POP is typically asymptomatic [11].

Clinicians dealing with women with POP might choose between surgery, pessary usage, pelvic floor muscle training, or observation. Nonsurgical approaches are usually chosen first by both clinicians and women. Improvement of symptoms and, for conservative management, minimization of prolapse progression are the main objectives of all treatments [9]. Although the patient’s preferences ultimately determine the course of therapy, people with symptomatic POP should be made aware that pessary usage is a feasible nonsurgical alternative. In case of unresponsiveness to non-invasive strategies, surgical correction of POP is often considered [1,3].

In order to provide a comprehensive evaluation of the clinical, surgical, instrumental, and prognostic aspects of the association between POP and LUTS, we divided this review into different sections. The main objective was to evaluate the newly studied and epidemiologic association between POP and LUTS; the grade of pop and severity of urinary symptoms; the role of urodynamics (UDS); and the influence of surgical and medical treatments on the symptoms and the patients’ quality of life.

## 2. Materials and Methods

**Figure 1:**
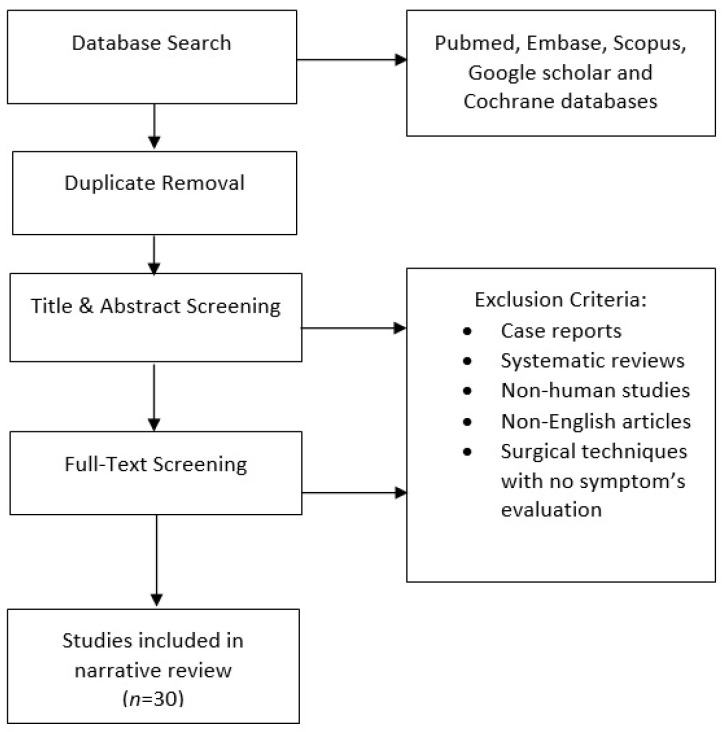
Summary of the search and screening methods applied in this narrative review.

A literature search was performed using the Pubmed, Embase, Scopus, Google Scholar, and Cochrane databases to find correlations between POP and LUTS, using as mesh terms “pelvic organ prolapse” and “lower urinary tract symptoms”. The literature review was conducted independently by two authors (GM and DL). Additional relevant articles that were cited in these original articles were added. Case reports, systematic reviews, non-human studies, and non-English articles were excluded. In addition, articles that focused on practical surgical techniques with no symptom evaluation were excluded. Figure 1 describes the search steps and screening procedure. In this narrative review, we focused on the topics in the subsequent sections:

## 3. What Is the Relationship between POP and LUTS?

Urinary problems, such as urgency, frequency, and difficulty of voiding, are prevalent in patients affected by uterine prolapse, although the link between prolapse and LUTS is unclear [12]. Cameron et al. evidenced a relationship between POP and LUTS, with a relative risk ranging from 1.2 to 5.8 [11]. It is unknown exactly why LUTS and POP occur together in a large percentage of women, but there is no plausible pathophysiological or anatomical explanation for why LUTS would produce POP [11]. There are, however, reasonable hypotheses for why POP could produce LUTS. POP is frequently associated with bladder outlet obstruction (BOO) and correlates with POP severity [13]. There is a consensus that POP-induced BOO may generate bladder alterations that result in overactive bladder (OAB) symptoms [13]. De Boer et al. [13] hypothesized three possible explanations for the correlation between POP and OAB:

- Neurological damage, with autonomic nerve and spinal micturition reflex;
- Alteration of the structure of the detrusor muscle;
- There is a the possibility that the stretching of the bladder causes stretch receptors in the urothelium to misfire and release neurotransmitters, such as acetylcholine and ATP [14,15]. Neurons detect neurotransmitters in the urothelium, which triggers the contraction of the bladder. However, many disorders may also share a common cause, including pelvic floor dysfunction, childbirth-related trauma, or ageing [16,17].

## 4. Is the POP Stage Related to OAB?

POP staging is currently reported using two major classifications, the Baden–Walker and the POP Quantification System (POP-Q) (Table 1).

For the purposes of the Baden–Walker system, the vagina is separated into six sections: two anterior, two superior, and two posterior. Using the hymen as point 0, each is given a score between 0 and 4 based on how much descent is present while the patient is exerting themselves to the fullest [18].

Nine locations in the vagina are measured by the POP-Q evaluation technique. The hymen serves as a baseline against which all other points are measured. The prolapsed organs are measured in millimeters to the hymen. The measurements are made while the person is in the dorsal lithotomy position and the Valsalva technique is being performed. The cervix, hymen, perineal body, total vaginal length, posterior vaginal wall, and posterior fornix are the anatomical landmarks employed. The numbers at the proximal and distal ends are recorded on a three-by-three centimeter grid. The proper stage of prolapse is determined from the data on the grid [19].

It is unknown if the severity of POP and OAB are related [11]. However, contrary to expectations, Burrows et al. [20] discovered that urgency and urgency urinary incontinence (UUI) occurred more frequently in women with less advanced POP. It was confirmed by Kowalski et al. in a recent study [21]. Another ultrasound-based study indicated that women with a lower grade of prolapse bladder develop UUI more frequently [22]. In contrast, Miranne et al. found that a higher percentage of women with a more advanced stage of POP demonstrated urodynamic detrusor overactivity (DO) (35%) compared to women with an earlier stage of POP [23]. The severity of POP is related to obstructive symptoms but not necessarily to other urinary symptoms. Anterior and apical vaginal compartment prolapse had a stronger link to OAB symptoms than to posterior vaginal compartment prolapse [24]. OAB syndrome is prevalent in the early stages of anterior vaginal wall prolapse, while posterior compartment prolapse may reduce its incidence [25].

## 5. Is LUTS Relief Possible after a POP Repair?

Surgical repair is not required to alleviate symptoms. At four months, women using a pessary had a 38% reduction in urgency and a 26% reduction in UUI [26]. In another study on women with UUI and POP, effective pessary fitting led to a 46% improvement in symptoms [27]. Pessaries have the potential to be helpful instruments in decision making prior to the beginning of a surgical procedure [11]. The patient’s LUTS were successfully treated with POP therapy, supporting the hypothesis that there is a connection between POP and LUTS [11].

Women who are concerned by their POP and have tried or refused nonsurgical therapies are candidates for surgical correction. There are several abdominal and vaginal surgical methods for treating POP [19]

The surgical management of POP depends on the localization of the defect. According to a vast survey, with or without the use of a synthetic graft, anterior colporrhaphy remained the most popular procedure for treating anterior compartment prolapse; vaginal hysterectomy with uterosacral suspension was used to treat uterine prolapse; posterior native tissue colporrhaphy was the most popular procedure for treating posterior compartment prolapse; and post-hysterectomy apical prolapse was repaired with abdominal sacrocolpopexy in 44% of cases [28].

In the past, a mesh material was employed in around one-third of urogynecological surgical operations for POP [29]. As many prosthetic operations, including urogynecological surgery, there have been documented cases of postoperative infection and migration problems with respect to prostheses [29].

## 6. Outcomes after Surgical POP Repair

De Boer et al. [13] collected 12 studies that examined OAB symptoms before and after POP repair. The duration of follow-up ranged from 2.5 to 60 months, and most studies reported a resolution rate of 90%. The RR of resolution was calculated by dividing the number of pre-operative symptoms by the number of post-operative symptoms. One trial revealed an RR of more than 1, with values ranging from 1.1 to 10.3 in 61% of patients [11,16]. Symptoms of OAB improve independently using the surgical method [11,30]. This is supported by Foster et al. [26], who showed how colpocleisis and reconstructive vaginal suspensions make no difference in OAB resolution one year after surgery. Following POP surgery, the majority of women who experience POP symptoms and have UI find that their UI is resolved or improved [31]. After correction, OAB symptoms improve independently from the POP severity [32].

## 7. POP Correction and OAB Improvement: Which Variables Influenced the Persistence of the Symptoms?

According to recent evidence, it is possible to identify clinical factors associated with the persistence of OAB symptoms following surgery. For example, in the current Cochrane review on POP surgical therapy, new or de novo OAB symptoms were reported in 12% of women in nine trials [33]. POP causes BOO, which leads to increased DO and OAB symptoms, but it may also cause permanent changes to the detrusor muscle, which may cause persistent bladder overactivity [11]. Patient factors that predicted persistent UUI included pre-operative urodynamic studies. Successful treatment of patients with DO and following POP surgery was the best therapeutic option for symptom improvement [34].

In another study, 24.5% of women with POP and DO showed persistent symptoms after prolapse surgical reduction (particularly with the mesh repair technique). At the same time, 3.5% of the patients had UDS evidence of DO only after surgery [35]. An age > 65 years, neurological diseases such as Parkinson’s, BOO, or increased PVR > 200 mL were associated with persistent or de novo DO [35]. Pre-operative OAB is a risk factor for OAB persistence after POP reduction, while age and sling placement correlate with de novo OAB. Increased BMI is associated with post-operative OAB [36]. Patients with severe pre-operative symptoms, neurologic disease, pelvic floor dysfunction, bladder neck obstruction, or severe anterior wall prolapse are at risk of post-operative voiding dysfunction [37]. Many patients diagnosed with UI after POP reduction had no more symptoms. UI degree and previous anti-incontinence surgery are predictors of the persistence of UI after POP reduction [38]. Liedl B et al. demonstrate that moderate-to-severe OAB complaints could be associated with high degree POP and symptom relief occurred after adequate surgical reconstruction [39]. According to these results, Malanowska et al. demonstrated that surgery, including laparoscopic lateral suspension in patients with POP and OAB, could help relieve urinary symptoms [40]. Ling-Ying Wu et al., in their study, also confirmed the positive role in improving storage symptoms after reconstructive surgery [30]. OAB improved significantly after surgical correction of POP, according to Johnson et al., but age was connected with the persistence of storage symptoms, such as urgency [16]. Päivi K Karjalainen et al. evidenced that surgical repair of POP could relieve OAB, particularly after reconstructing anterior and apical compartments. They showed a correlation between apical and anterior POP and the severity of OAB symptoms. These patients had better relief after reconstructive surgery [24]. Ahmed M Tawfeek et al. performed a pre-operative and post-operative UDS in patients with LUTS. They showed that urinary symptoms improved after surgery, and DO was associated with the persistence of voiding symptoms [41].

Taking these findings together, even if the surgical correction of POP can improve OAB symptoms in most women, the presence of independent risk factors, including Parkinson’s disease or other neurological pathologies, DO, advanced age, or increased BMI, might lead to the persistence of symptomatology even after successful reconstructive surgery.

## 8. Medical Treatment of LUTS?

According to OAB guidelines, antimuscarinics and 3-adrenoceptor agonists are second-line therapy for OAB [42]. The efficacy of medical therapy for OAB treatment has been limited and may also be associated with undesirable side effects. Following POP surgery, it can be used temporarily or as a therapy for persistent or new-onset OAB [11].

## 9. Urodynamics and POP Repair

Given the weak correlation between DO and UUI, preoperative DO is not always a good predictor of UUI after surgery. This finding aids in the prediction of postoperative outcomes, so routine preoperative pressure-flow UDS in women undergoing POP repair is not warranted [11]. Pressure-flow UDS could be proposed in patients with hydronephrosis, neurological disease, recurrent urinary tract infections (UTIs), and particularly for pre-surgery management [43]. If UDS is performed following the AUA/SUFU guidelines [44], the POP should be lowered to test detrusor dysfunction, and this procedure may differentiate between BOO and detrusor underactivity (DU). UDS should be performed on a subset of POP patients. This test can help us counsel POP patients with voiding symptoms, but it cannot provide additional information for patients with POP and UI [45]. UDS had no significant impact on preoperative management or counselling in POP surgery if occult stress urinary incontinence was not the indication for preoperative testing in women undergoing POP surgery [46]. Research, however, argues for UDS before surgery as a screening tool to prevent unnecessary concurrent continence treatments [47].

## 10. Sexuality after POP Repair

It is critical to incorporate sexual health into therapeutic practice. Evaluation of anatomical anomalies, lower urinary tract function, and anorectal function are sometimes given greater consideration in women with pelvic floor issues than sexual function.

The majority of women who have symptoms of POP and LUTS are still engaged in sexual activity. The possibility that female sexual dysfunctions will be identified may rise with the use of questionnaires and sexuality scales to help women and health care providers address sexual issues. The only questionnaires currently validated and created particularly to measure female sexual function in women with urine incontinence and/or pelvic organ prolapse are the Pelvic Organ Prolapse Incontinence Sexual Questionnaire (PISQ) and the PISQ-12-IR (IUGA-Revised). The PISQ-12-IR also enables examination of the results for urogynecologic care-requiring women who are not sexually active [48].

Ageing and menopause are linked to decreased sexual activity. However, the effects of surgery to cure LUTS and POP on female sexual function are still being debated.

Li et al. conducted a prospective observational study to determine the prevalence of female sexual dysfunction, relevant risk factors, and the effects of pelvic floor surgery in women who have POP, stress urinary incontinence, or both [48].

Prior to and following surgery, sexual activity and sexual function were examined together with potential risk factors. The Female Sexual Function Index and the PISQ-12 are two validated questionnaires that were used to assess sexual function. They showed that age and postmenopausal status were factors in the absence of sexual activity prior to surgery. Similarly, sexual dysfunction was linked with advancing age. Overall, there was no difference in the PISQ-12 score before and 12 months after surgery. Lubrication of the vagina was an unrelated feature that was linked to an improvement in sexual function following surgery. The improvement in the quality of sexual life following surgery was significantly impacted by menopause [48].

Similarly, Mattsson et al. evaluated the impact of female POP surgery on sexuality, patient satisfaction, and health-related quality of life; they also identified outcome predictors. Over a two-year follow-up period, 7 out of 10 patients with POP surgery reported an improvement in sexual habits and health-related quality of life; patient satisfaction was high. The most reliable indicators of improvement were vaginal bulging and apical prolapse beyond the hymen. They also highlighted that patients should be urged to give up smoking in order to prevent a negative outcome [49].

However, dyspareunia occurred in 26% of 53 women after posterior colporrhaphy and in 38% of women with both Burch colposuspension and posterior colporrhaphy, according to Weber et al.’s analysis of 81 women who underwent surgery for prolapse or urinary incontinence [50]. There were no site-specific procedures employed for posterior repair. Using general or non-pelvic floor disorder-specific questionnaires, others have discovered stable or declining sexual function. It is possible that some partners are concerned that sexual activity would affect the postoperative results because some patients had lower scores on the partner-related items. Therefore, it is likely necessary to know about favorable sexual outcomes [50].

According to Glavind et al. [51] the majority of women who receive surgical treatment for various forms of POP employing native tissue restoration and “site-specific” procedures report an improvement in their sexual lives thereafter. Preoperative urine incontinence was cured or improved in the majority of individuals [51].

In terms of how surgery effectively ameliorates sexual health rather than a different approach, several studies have been conducted.

Kinjo et al. compared the effectiveness of vaginal pessaries and modified transvaginal mesh (TVM) surgery in women with symptomatic POP [52].

They examined 130 symptomatic POP patients who had received either vaginal pessaries or modified TVM. Lower urinary tract, bowel, and sexual problems were evaluated together with the prolapse-related quality of life (QOL) using the prolapse QOL questionnaire. All questionnaires were completed before and a year after the therapy. One year following therapy, the prolapse and voiding symptoms as well as all the prolapse-related QOL categories were considerably better in the pessary group, with the exception of the personal connections and sleep/energy. The modified-TVM group saw a considerable improvement in all QOL categories as well as prolapse, urine storage, voiding, bowel, and sexual problems. They concluded that the vaginal pessary and modified TVM surgery both successfully cured prolapse and voiding symptoms and enhanced the majority of prolapse-related QOL variables. Modified TVM surgery was superior to pessary therapy in terms of relieving urine storage, bowel, and sexual complaints. However, compared to pessary insertion, modified TVM appeared to place the organs more effectively to enhance bowel, sexual, and bladder function [52].

Similarly, Van der Vaart et al. reported that surgery had a better effect on sexual function in sexually active POP patients than pessary treatment [53]. The improvement is mostly due to the POP symptoms’ diminished influence on sexual performance. Sexually active women who declare that POP-related symptoms are bothering their sexual performance should be advised that surgery produces more notable relief. A superior course of therapy could not be proven for sexually inactive women. In contrast to pessary therapy, considerably more NSA women underwent surgery than pessary therapy, despite the fact that the differences in PISQ-IR scores between the two groups of NSA women were not statistically different. The patient must be given this information, which includes weighing the advantages and disadvantages of the surgery and the pessary, in order to make a balanced choice [53].

However, when performing surgery for POP, to avoid useless damages on sexual health, several considerations should be considered.

Giving realistic expectations of surgical results is crucial before undergoing pelvic floor disease surgery. In order to create a baseline, it is crucial to evaluate the patient’s sexual orientation, attitude toward sexual activity, the effect of pelvic disease on sexual relations, the quality of orgasms, and desire. To prevent subsequent disappointment, patient expectations of the effects of pelvic floor surgery should be investigated and appropriate goals should be set. In the absence of pelvic disease, pre-existing sexual issues might indicate a worse than ideal outcome [54].

Sexual dysfunction in SUI patients may be exacerbated by damage to the vaginal innervation on the anterior and distal parts of the vaginal wall. To prevent such damage, excessive dissection should be avoided. Synthetic tape should not be placed too superficially while performing a mid-urethral sling surgery since this might cause discomfort during penetration and dyspareunia if the mesh gets exposed. When performing a mid-urethral sling surgery, excessive lateral positioning of the needles should be avoided in order to avoid serious vascular damage [54].

Vaginal length should be preserved whenever feasible in women having vaginal hysterectomy for prolapse or an anterior repair, and extensive vaginal mucosal excision should be avoided. Sexual function was unaffected by the vaginal cuff closure technique (vertical vs. horizontal closure). It is crucial to preserve introital caliber and vaginal length while doing a posterior repair. A suitable caliber would be 8–11 cm long and it should be possible to accommodate two or three fingers during vaginal reconstruction surgery. The likelihood of developing dyspareunia was significantly enhanced when an anterior colporrhaphy was combined with a posterior vaginal wall repair; therefore, there may be a purpose for doing the two operations independently when practical [55].

This would also suggest that in sexually active women, a prophylactic posterior repair should be avoided at the time of an anterior repair. A levator plication should be avoided in sexually active women because it results in a constriction ring inside the vagina. Whenever feasible, surgical methods should be used to prevent constriction of the vaginal introitus [55].

To maintain sexual function in sexually active women with a vaginal vault prolapse, a sacrocolpopexy should be recommended instead of a sacro-spinous fixation [54].

## 11. Conclusions

The link between OAB and POP in women is unclear. BOO due to POP and accompanying bladder muscle alterations appear to be the most probable explanation for how POP might induce OAB and explains why POP repair improves OAB symptoms in the majority of patients. This argument contradicts the idea that severe POP (which generates more BOO) does not induce worse OAB. A considerable proportion of women do not have OAB resolution following POP surgery, potentially due to permanent bladder alterations due to long-term BOO, or perhaps they are different conditions in particular women since OAB occurs without POP or BOO in many women. POP reduction should also be the first-line treatment for DU in women with coexisting POP, considering the limited treatment choices for this disorder. As this may be a unifying diagnosis and a single surgery might solve both conditions, it would appear wise to provide POP reduction surgically or with a pessary as the primary treatment choice for all women with OAB and POP. As the presence of DO, DU, or BOO does not alter surgical planning, UDS are not required before surgery unless the patient has a history of neurological disease, hydronephrosis, or recurrent UTIs. However, simple cystometrics with POP reduction are required prior to surgery to assess for occult SUI and to counsel patients on their risk of postoperative SUI to assist them in deciding whether to undergo a concomitant sling procedure. Women with bladder trabeculation, longer duration of OAB symptoms, age > 65 years, neurological disease, higher PVR > 200 mL, and greater degrees of BOO have a reduced likelihood of OAB remission following POP surgery. However, none of these risk factors significantly reduced the likelihood of resolution. Thus, they are not indicators against giving POP correction. The potential impact on sexual function should be discussed and used to guide decision making when counseling women with a prolapse or incontinence, especially prior to surgical treatment. It is essential to test sexual function before surgery in order to find any underlying sexual issues. This causes less disappointment when desired outcomes are not achieved and allows for more reasonable expectations from the therapies that are accessible. According to the most recent and reliable research, women should obtain counseling and management. It is important to understand the benefits and drawbacks of the procedure and how it can affect sexual function in sexually active women who are having pelvic floor surgery.

## Figures and Tables

**Table 1:** POP grading according to Baden–Walker and POP-Q classification systems.

Grade	Baden–Walker	POP-Q
0	Normal position for each respective site	No prolapse
1	Descent halfway to the hymen	Greater than 1 cm above the hymen
2	Descent to the hymen	1 cm or less proximal or distal to the plane of the hymen
3	Descent halfway past the hymen	Greater than 1 cm below the plane of the hymen, but protruding no further than 2 cm less than the total vaginal length
4	Maximum possible descent for each site	Eversion of the lower genital tract is complete

## Data Availability

No new data were created or analyzed in this study. Data sharing is not applicable to this article.
